# Fractional hitting sets for efficient multiset sketching

**DOI:** 10.1186/s13015-024-00268-0

**Published:** 2025-02-08

**Authors:** Timothé Rouzé, Igor Martayan, Camille Marchet, Antoine Limasset

**Affiliations:** 1 G5 - SeqBio, Institut pasteur, Université Paris Cité, 75724 Paris, France; 2 UMR9189 CRIStAL, Univ Lille, CNRS, Centrale, 59000 Lille, France; 3 Sorbonne Université, Collège Doctoral, 75005 Paris, France

**Keywords:** *k*-mer, Subsampling, Sketching, Jaccard, Containment, Metagenomics

## Abstract

The exponential increase in publicly available sequencing data and genomic resources necessitates the development of highly efficient methods for data processing and analysis. Locality-sensitive hashing techniques have successfully transformed large datasets into smaller, more manageable sketches while maintaining comparability using metrics such as Jaccard and containment indices. However, fixed-size sketches encounter difficulties when applied to divergent datasets. Scalable sketching methods, such as sourmash, provide valuable solutions but still lack resource-efficient, tailored indexing. Our objective is to create lighter sketches with comparable results while enhancing efficiency. We introduce the concept of Fractional Hitting Sets, a generalization of Universal Hitting Sets, which cover a specified fraction of the *k*-mer space. In theory and practice, we demonstrate the feasibility of achieving such coverage with simple but highly efficient schemes. By encoding the covered *k*-mers as super-*k*-mers, we provide a space-efficient exact representation that also enables optimized comparisons. Our novel tool, supersampler, implements this scheme, and experimental results with real bacterial collections closely match our theoretical findings. In comparison to sourmash, supersampler achieves similar outcomes while utilizing an order of magnitude less space and memory and operating several times faster. This highlights the potential of our approach in addressing the challenges presented by the ever-expanding landscape of genomic data. supersampler is an open-source software and can be accessed at https://github.com/TimRouze/supersampler. The data required to reproduce the results presented in this manuscript is available at https://github.com/TimRouze/supersampler/experiments.

## Introduction

The field of genomics has exploded in recent years, driven by the availability of cheap and easy sequencing data generation. The Sequence Read Archive (SRA) is a vast and to some extent under-exploited goldmine of genomic data, containing an enormous amount of genetic information. However, one of the biggest challenges in utilizing this data is the lack of efficient indexing and querying tools. The GenBank database, for example, already contains 1.2 million bacterial genomes, totaling over 5 Terabases of data. In the face of such vast genetic information, a crucial need is to promptly and precisely determine the most similar (or contained) known entry for a given query document (assembled or unassembled reads). Specifically, in this work we focus on the metagenomic assessment problem, which entails characterizing microbial communities in a specific environment using DNA sequencing data and potentially large amounts of reference entries. The complexity and diversity of the data, which contains sequences from multiple genomes, presents significant challenges.

In the metagenomic context, traditional alignment-based methods such as BLAST are increasingly computationally prohibitive due to the sheer number of potential targets for metagenome mapping. A spectrum of alignment-free techniques based on *k*-mer content have emerged as a viable alternative, with different tradeoffs. On one side of the spectrum, exact *k*-mer indexing offers linear query time [1–3], but may be too memory-intensive for large-scale applications. Probabilistic structures, when applied to large queries (on the order of kilobases), enable more scalable indexing at the expense of a random false positive rate [4, 5].

Using large enough query data allows for handling false positive noise since it remains significantly lower than the required matching signal (e.g., 70% of queried *k*-mers present in the document). It means false positives are not a real issue if query size is adequate. For extensive queries at the Megabase level, the signal strength is sufficiently robust, eliminating the need to consider all *k*-mers and enabling sublinear query time. On the other side of the spectrum, fixed-size sketches like Minhash [6], Hyperloglog [7], and Hyperminhash [8] have been effectively used for large-scale collection comparison [9–13]. However, they are ill-suited for divergent documents in terms of content or size, a critical limitation considering metagenomic samples typically comprise many organisms with amount of distinct *k*-mers varying by orders of magnitude.

Scaled sketches, whose size scales linearly with input size, have demonstrated better resilience to such issues. sourmash [14], which implements scaledminhash [14] and Fracminhash [15], efficiently approximates containment and Jaccard indexes even for documents with size disparities spanning several orders of magnitude. sourmash’s simplicity is one of its key strengths: it stores each uniformly selected fingerprint as a 32-bit integer and compares them using a dictionary. An observation is that this technique is generic and can be applied to any type of data. Therefore, computational and memory requirements could benefit from customized selection techniques and index structures.

To this end, we propose capitalizing on the ability to represent overlapping *k*-mers with a low number of bits per *k*-mer using a *Spectrum Preserving String Set* [16]. The challenge we face is optimizing the overlap of chosen *k*-mers to achieve maximum space efficiency. To address this, we build upon the concept of super-*k*-mers [17], which are sequences of *k*-mers sharing a common selected *m*-mer called minimizer [18]. Universal Hitting Sets (UHS [19]) methods aim to design optimized *m*-mer selection schemes that covers all *k*-mers while minimizing the density of selected positions. However, our application does not require complete coverage of the *k*-mer space. Therefore, we introduce *Fractional Hitting Sets* that encompass a near-uniform selected fraction of the *k*-mer space. We conduct a study on the achievable density in relation to the selected fraction and present a straightforward minimizer selection scheme that closely approaches optimal bounds. We implement this scheme in a tool called supersampler. The storage of enhanced super-*k*-mer sequences, partitioned by minimizers, facilitates space and time-efficient *k*-mer set comparisons. Our evaluation reveals that supersampler significantly reduces resource usage compared to sourmash while maintaining similar results. Overall, this work presents a promising approach to making large-scale genomic data more accessible and manageable.

## Preliminaries

This paper presents results on finite strings on the DNA alphabet $\Sigma =\{A,C,G,T\}$, we use $\sigma$ to denote the size of the alphabet. We consider two input multisets of strings longer or equal to *k*, $S_A$ and $S_B$. These multisets can in practice be read sets from sequencing experiments or genome sequences. We call *k*-mers strings of size *k* over strings of the input sets. $A=\{x_0, x_1, \ldots , x_{n-1}\}$ is the set of distinct *k*-mers from $S_A$ and $B=\{y_0, y_1, \ldots , y_{n-1}\}$ is defined similarly for $S_B$.

The metrics to estimate the similarity between two sets are later defined in Set comparisons onwards.

We first state important definitions that will be used to introduce a first contribution of the paper: in section Fractional hitting sets we present the advantages of a sampling process that depends on minimizers instead of *k*-mers hashed values. Then in section Sketching technique in supersampler we describe our second contribution, a method for indexing the sampled elements and comparing them in an efficient manner for set and multiset similarity estimation.

*Minimizers and super*-*k*-***mers***

### Definition 1

(**minimizer**) Given $m < k$, a total order $\mathcal {O}$ on $\Sigma ^m$ and a *k*-mer *u*, the minimizer of *u* is the smallest *m*-mer of *u* according to $\mathcal {O}$.

The way minimizers are selected is referred to as a minimizer scheme. From now on, $\mathcal {O}$ is defined on integers by hashing *m*-mers using a random hash function *h* and minimizers are selected by choosing the smallest hash value. We assume that the hash function is chosen such that the hashes are independent and uniformly distributed. We use $w = k - m + 1$ to denote the number of *m*-mers inside a *k*-mer.

### Definition 2

(**super-***k***-mer**) A super-*k*-mer is a maximal substring of a string *s* ($|s|\ge k$) in which each consecutive *k*-mers have the same minimizer.

Spectrum Preserving String Sets [16] are an efficient *k*-mer encoding made of a collection of strings whose property is to spell exactly the initial set of *k*-mers, usually exploiting the fact that *k*-mers share overlaps.

Super-*k*-mers are an interesting *Spectrum Preserving String Set* because a super-*k*-mer containing *x* *k*-mers is composed of $k+x-1$ bases which incur a cost of $\frac{2(k+x-1)}{x}$ bits per *k*-mer. Therefore longer super-*k*-mers provide lighter representation as opposed to classic *k*-mer representation by simply avoiding to repeat an increasing number of nucleotide while super-*k*-mer size increases.

By omitting repeated minimizers inside *k*-mers, the first *k*-mer of the longest possible super-*k*-mers have their minimizers as a suffix. Equally, the last *k*-mers of these super-*k*-mers have their minimizers as a prefix.

### Definition 3

(**maximal super-***k***-mer**) Let *s* ($|s|\ge k$) be a string and *v* a super-*k*-mer of *s*. Let $i_m$ be the first position of the minimizer on *s*. *v* is a maximal super-*k*-mer iff *v* starts at position $i_m + m - k$ in *s* and *v* ends at position $i_m + k - 1$ in *s*. It follows that *v* has a length of $2k-m$ and contains $w=k-m+1$ *k*-mers.

**Fig. 1:**
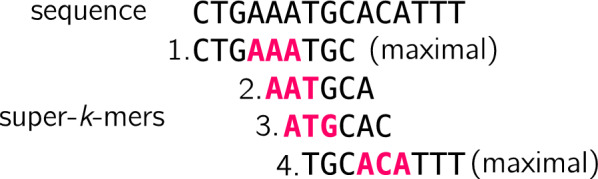
Super-*k*-mers extracted from a sequence for $k=6, m=3$. Minimizers are shown in pink, here we use the lexicographic order instead of hashing minimizers for the sake of the simplicity. Super-*k*-mers 1 and 4 are maximal (they contain respectively *k*-mers $\{CTGAAA, TGAAAT, GAAATG, AAATGC\}$ and $\{TGCACA, GCACAT, CACATT, ACATTT\}$, while 2 and 3 are not (and contain respectively *k*-mers $\{AATGCA\}$ and $\{ATGCAC\}$

Examples of regular and maximal super-*k*-mers are shown in Fig. 1. Since maximal super-*k*-mer are the most space-efficient, our approach aims to rely on long super-*k*-mers (ideally maximal) in order to have a compact encoding of the *k*-mer sketch. As mentioned in [20] (see the proof of theorem 3), the proportion of maximal super-*k*-mers approaches $\frac{1}{4}$ for a large *k*.

*Density and universal hitting sets* Since every minimizer corresponds to one super-*k*-mer, the proportion of *m*-mers chosen as minimizers is exactly the inverse of the average length of super-*k*-mers. This proportion, which quantifies the sparsity of a minimizer scheme, is referred to as the *density*.

### Definition 4

(**density of a minimizer scheme**) The density of a minimizer scheme is the expected number of selected minimizers divided by the total number of *m*-mers. The density factor is equal to the density multiplied by $w + 1$.

The density of a minimizer scheme is lower bounded by 1/*w* since each *k*-mer contains *w* *m*-mers and one of them must be selected as a minimizer. It is known that the expected density of a minimizer scheme based on a random ordering is $2 / (w+1)$, and that minimizer schemes cannot have a density below $1.5 / (w+1)$ [21]. More generally, *m*-mers selection scheme able to cover every *k*-mer are called *universal hitting sets*.

### Definition 5

(**universal hitting set or UHS**) A set $\mathcal {U}\subseteq \Sigma ^m$ is defined as a Universal Hitting Set (UHS) if every sequence of *w* consecutive *m*-mers has an element contained in $\mathcal {F}$.

Note in particular that the set of all minimizers of $\Sigma ^k$ forms a UHS. Recent publications introduced different methods based on UHS to bring the density below $2 / (w+1)$ and closer to the $1.5 / (w+1)$ barrier [22, 23]. Thus, a question that naturally arises is: can we cross this barrier by relaxing some constraints on the selection scheme?

## Fractional hitting sets

In this section, we introduce the concept of *fractional hitting sets*, which are a relaxation of universal hitting sets. These sets are designed to cover a fraction *f* of the *k*-mer space on expectation.

### Definition 6

(**fractional hitting set or FHS**) Given $f \le 1$, a set $\mathcal {F} \subseteq \Sigma ^m$ is a Fractional Hitting Set (FHS) if a fraction at least *f* of the sequences of *w* consecutive *m*-mers have an element contained in $\mathcal {F}$.

To avoid selection bias in practice, we aim to ensure that *m*-mers are selected randomly and have an equal chance of being chosen by using a random hash function that uniformly distributes the *m*-mers over $\llbracket 1, \sigma ^m \rrbracket$.

We introduce a simple probabilistic method to build such fractional hitting sets by selecting minimizers with a hash smaller than a certain threshold. We call such selected minimizers *small minimizers*. Note that any method selecting a fraction of the minimizers hashes would be suitable here.

### Definition 7

(**small** *m***-mer**) Given a fixed threshold $t \in \llbracket 1, \sigma ^m \rrbracket$, we say that a *m*-mer is small if its hash is below *t*. We denote by $\mathcal {S}$ the set of small *m*-mers, and $p = \frac{t}{\sigma ^m}$ the probability that a *m*-mer is small.

From *p* we can derive the proportion of covered *k*-mers.

### Property 1

Given $t \in \llbracket 1, \sigma ^m \rrbracket$ and $p = \frac{t}{\sigma ^m}$, the expected fraction of *k*-mers with distinct *m*-mers containing a small *m*-mer is

$$f = 1 - (1 - p)^w$$

where $w = k - m + 1$ and *p* is the probability that a *m*-mer is small.

### Proof

Given a *k*-mer with $w$ distinct $m$-mers $x_1, \dots , x_{w}$,

$$\mathbb {P}(\forall i \in \llbracket 1, w \rrbracket , h(x_i)> t) = \prod _{i=1}^{w} \mathbb {P}(h(x_i)> t) = (1 - p)^w$$

because the hashes of distinct *m*-mers are independent, so

$$f = \mathbb {P}\left( \min _{i \in \llbracket 1, w \rrbracket } h(x_i) \le t \right) = 1 - (1 - p)^w$$

$$\square$$

Note that this property is valid for *k*-mers with distinct *m*-mers; *k*-mers with duplicated *m*-mers (i.e. *k*-mers containing repetitions) have a lower coverage since they have less candidates for small minimizers. Fortunately, as shown in [22] (see lemma 9), the proportion of *k*-mers with duplicated *m*-mers is negligible for a sufficiently large *m* ($> (3 + \varepsilon ) \log _\sigma w$). Thus, our selection method is uniform for *k*-mers with distinct *m*-mers, and almost uniform in the entire *k*-mer space.

Conversely, if we want a given fraction *f* of the *k*-mers to be covered, the threshold should be chosen as

1 $$\begin{aligned} t = \left[ 1 - (1 - f)^{1/w} \right] \cdot \sigma ^m \end{aligned}$$

Related to this, let us define the subsampling rate as $s = \frac{1}{f}$. For instance, a desired subsampling rate of 1000 will give $f=\frac{1}{1000}$.

*Density of small minimizers* We showed that selecting *k*-mers with a small minimizer induces an FHS. By considering the usual definition of density (from Definition 4) for this scheme (which covers a fraction *f* of all *k*-mers), we obtain the following bound (proven in supplementary materials):

### Theorem 1

Given $f \le 1$ and $t = \left[ 1 - (1 - f)^{1/w} \right] \cdot \sigma ^m$, assuming $m> (3 + \varepsilon ) \log _\sigma w$, the expected density of small minimizers in a random sequence is upper bounded by

$$\frac{2 f}{w + 1} + o(1/w)$$

At first glance, the results may be surprising, as the density is smaller than the lower bound of 1/*w* for $f < 1/2$ and can approach zero. This is because some *k*-mers may not contain any small minimizers and are therefore not covered, and the proportion of such *k*-mers increases as *f* approaches 0. However, it is worth noting that this bound does match the $2/(w+1)$ density when $f=1$ (i.e., when every *k*-mer is covered).

To obtain a more meaningful metric, we can compute the density on the fraction of the sequence that is covered, instead of the entire sequence. With this approach, we obtain the following theorem, which has been proven in the supplementary materials:

### Theorem 2

**(restricted density)** If we restrict to sequences in which every *k*-mer contains a small minimizer, then given $f \le 1$ and $t = \left[ 1 - (1 - f)^{1/w} \right] \cdot \sigma ^m$, assuming $m> (3 + \varepsilon ) \log _\sigma w$, the expected density of small minimizers is upper bounded by

$$2 \cdot \frac{f + (1 - f) \ln (1 - f)}{f^2 (w+1)} + o(1/w)$$

Although less intuitive than the previous one, this result provides valuable insights into the density within the covered portion of the sequence. As shown in Fig. 6 (see supplementary materials), the associated density factor ranges from 2 when $f=1$ (consistent with existing results) to 1 when $f=0$. Therefore, as *f* approaches 0, we can approach the optimal density.

*Proportion of maximal super*-*k*-***mers***

Although measuring the density provides an overview of the average length of super-*k*-mers, it does not indicate how many of them are maximal (i.e., of length $2k-m$). The following result (proven in the supplementary materials) answers this question:

### Theorem 3

Given $f \le 1$ and $t = \left[ 1 - (1 - f)^{1/w} \right] \cdot \sigma ^m$, the average proportion of maximal super-$k$-mers (with respect to all super-$k$-mers) built from small minimizers in a random sequence is given by

$$\begin{aligned} \left[ \left( 1 - \frac{1}{w} \right) \frac{f}{1 + f} \right] ^2 + \frac{1 - f(1 - 2/w)}{1 + f} \end{aligned}$$

Note that this result generalizes theorem 2 from [20], which corresponds to $f = 1$. As shown in Figure 15b (see supplementary materials), the proportion increases towards 100% as *f* approaches 0.

*Improving the density of fractional hitting sets using UHS* This effect is more pronounced for smaller values of *f*. This observation raises a natural question: what is the lowest achievable density for a given *f*? Since universal hitting sets (UHS) with a density lower than 2 have been proposed for $f=1$, it is possible that they may also improve the density for smaller *f* values by considering only the *m*-mers selected by the UHS as potential minimizers.

### Theorem 4

Given a UHS $\mathcal {U}$ with density $d_\mathcal {U}$, $f \le 1$ and $t = \left[ 1 - (1 - f)^{1/w} \right] \cdot \sigma ^m$, assuming $m> (3 + \varepsilon ) \log _\sigma w$, the expected density of small minimizers selected from $\mathcal {U}$ (that is, $\mathcal {S}\cap \mathcal {U}$) in a random sequence is upper bounded by

$$f \cdot d_\mathcal {U}+ o(1/w)$$

The proof is given in supplementary materials. Note that this result generalizes theorem 1 since the UHS of minimizers selected using a random ordering has a density of $2/(w+1)$ [21].

## Sketching technique in supersampler

### Supersampler’s sketch construction

#### Definition 8

(**supersampler’s sketch**) Given a sequence *S*, each super-*k*-mer whose minimizer’s hash is lower or equal to a threshold *t* is selected, and all its surrounding *k*-mers are kept in the sketch as a super-*k*-mer. A supersampler sketch can therefore be represented as a super-*k*-mer set.

In sourmash, *k*-mers are represented as integer fingerprints through hashing (using a default of 32-bit). This approach reduces space requirements compared to employing 2*k* bits per *k*-mer, but it also introduces the potential for false positives due to hash collisions. However, the false positive rate is exponentially low, depending on the fingerprint size, which allows for efficient control.

**Fig. 2:**
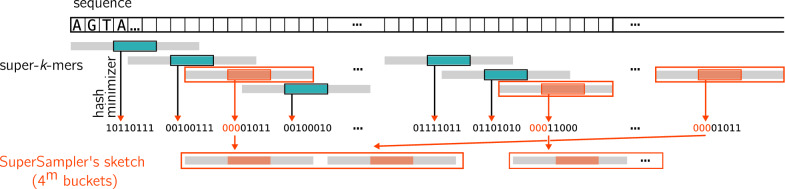
supersampler’s sketching strategy. In order to build sketches, supersampler computes super-*k*-mers over the input sequence. Fingerprints are associated with each super-*k*-mer by hashing their minimizers to an integer, hence an integer per super-*k*-mer. Super-*k*-mers associated to sufficiently low integers are kept in the sketch. Super-*k*-mers are put into partitions according to their minimizer

In contrast, supersampler explicitly stores *k*-mers as super-*k*-mers, offering two significant advantages over the conventional method: First, the fingerprints are represented exactly, eliminating false matches and enabling the output of shared *k*-mers when they are of interest to users. Second, this technique enables a more space-efficient representation of *k*-mers, typically requiring less than the 32-bit per *k*-mer space cost of sourmash. Figure 2 illustrates an example of sketch construction in supersampler. In the following sections, we propose a model to evaluate the space efficiency of a supersampler sketch.

In the regular case with hashed minimizer with a density factor of 2 we can expect $(k-m+1)/2$ *k*-mers per super-*k*-mers [20]. This results in a mean super-*k*-mer length of $(3k-m-1)/2$ bases. We can give a lower bound of the cost in bit per *k*-mer to encode such super-*k*-mers:

$$\frac{2(3k-m-1)}{k-m+1}$$

However we need to encode the length of each super-*k*-mer to avoid considering artefactual *k*-mers created by two successive super-*k*-mers so we can add $\log _2(k-m+1)$ bits per super-*k*-mer (encoding the number of *k*-mers). This leads to a bits per *k*-mer ratio of

$$2 \cdot \frac{3k - m - 1 + \log _2(k - m + 1)}{k - m + 1}$$

In practice we use the formula (1) to select a *k*-mer fraction chosen by the user. As a side effect, since we used an FHS, selected super-*k*-mers are longer than those selected by regular hashed minimizer scheme as our hitting set provides a lower density. Importantly for low selected fraction a very large proportion of super-*k*-mers are maximal. This property is of prime interest because maximal super-*k*-mers can be efficiently encoded for two reasons. First they are all of the same size so we do not need to encode their respective length or any kind of separator. Second, they represent $2k-m$ bases encoding for $k-m+1$ *k*-mers, they provide a lower bits per *k*-mers ratio

$$\frac{2(2k-m)}{k-m+1}$$

### Partitioned sketches

Minimizers naturally splits the super-*k*-mer space into $\mathcal {O}(4^m)$ partitions. Since only a subset of the minimizer space is selected, a smaller number of partitions are actually considered. supersampler relies on the fact that super-*k*-mers are centered around a shared minimizer to build a partitioned sketch. In practice, we examine all non-empty selected partitions, each storing a minimizer with their corresponding super-*k*-mers independently. This strategy offers several crucial advantages. First, when encoding maximal super-*k*-mers, we know the position of the minimizer within each super-*k*-mer. By storing the minimizer sequence once in the partition, we can omit it in all maximal super-*k*-mers. This results in an even lower bit per *k*-mer ratio:

$$\frac{4(k-m)}{k-m+1}$$

**Fig. 3:**
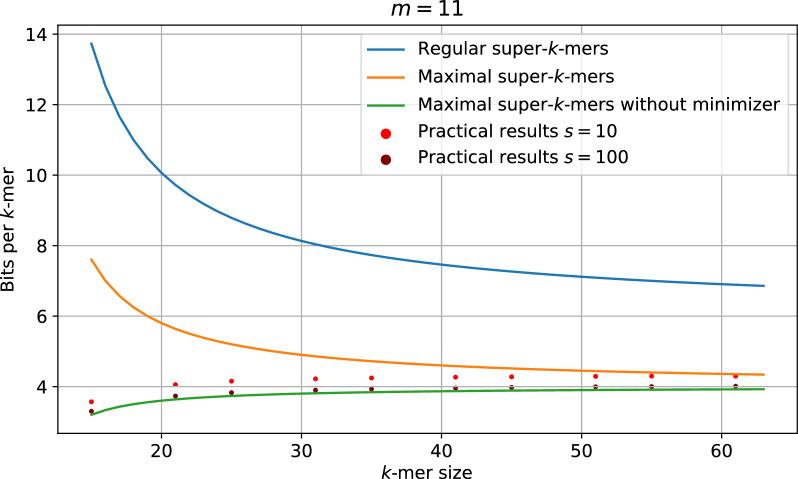
Theoretical space cost of different encodings in bits per *k*-mer according to the *k*-mer size along with practical space usage of super-sampler sketches on random sequences

Fig. 3 shows the space cost of the different encoding: super-*k*-mer, maximal super-*k*-mer and partitioned super-*k*-mer according the the minimizer size along with the actual performances of supersampler.

This mechanism, by enhancing space efficiency, implies that storing larger *k*-mers as maximal super-*k*-mers helps reducing memory usage. When comparing document sketches, matching *k*-mers between documents are necessarily found in the same partition, so only a given partition is needed in memory at a time. For sufficiently large *m*, such partitions should be orders of magnitude smaller than the total amount of fingerprints, as the expected partition size decreases exponentially with *m*. This partitioning technique also allows for substantial speed-ups, notably in sketch comparison time, which are discussed later on.

*Abundance Filtering* When working with raw read sets, a practical feature is the ability to filter out low-abundance $k$-mers, which are likely attributable to sequencing errors. Notably, sourmash lacks this feature and requires users to handle such filtering independently. Our approach is both user-friendly and efficient, as we store only fingerprints abundances, a process considerably less resource-intensive than standard *k*-mer counting.

However, a potential issue arises when applying abundance filtering: certain *k*-mer within a chosen super-*k*-mer might be excluded, effectively “breaking” them. To address this, we initiate an “assembly” phase for all *k*-mers not already part of a maximal super-*k*-mer. The goal is to produce as many maximal super-*k*-mers as possible, while minimizing non-maximal super-*k*-mers. We commence with *k*-mers having minimizers as suffixes and attempt to extend them, using a greedy method, until a maximal super-*k*-mer is obtained. When no more maximal super-*k*-mers can be constructed, we aim to build the largest possible sequences in a greedy fashion to minimize the total base number similarly to simplitigs [24].

### Set comparisons

**Fig. 4:**
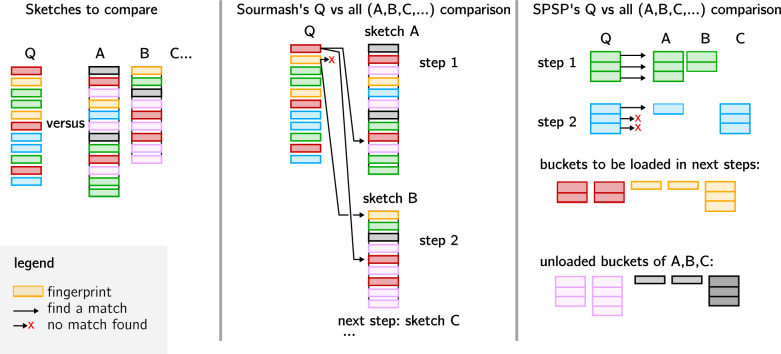
How supersampler and sourmash perform their respective sketch comparison. Colored rectangles represent *k*-mers. Those sharing the same color are sharing a common minimizer. In supersampler sketches, *k*-mers sharing their minimizers are stored in the same partition. In this example, we discuss the comparison of one document against a collection, although other use cases can be inferred. supersampler is capable of skipping certain partitions that are not relevant to the query. By focusing on smaller sub-parts of the collection one at a time, supersampler effectively improves practical performance and reduces memory usage

*Jaccard and containment indexes* For two finite, non empty sets of *k*-mers *A* and *B*, the Jaccard index [6] gives a measure of the similarity *A* and *B* by comparing the relative size of the $A \cap B$ intersection over the $A \cup B$ union,

$$0 \le J_{A,B} = \frac{|A \cap B|}{|A \cup B|} \le 1$$

The containment index *C* of the previously defined sets A and B measures the relative size of $A\cap B$ intersection over the size of A, i.e., the proportion of distinct *k*-mers of A that are present in B.

$$0 \le C_{A,B} = \frac{|A \cap B|}{|A|} \le 1$$

supersampler’s sketch comparison, in line with previous works, produces an estimator for both Jaccard and containment indexes.

*Speed up sketch comparisons* Unlike sourmash that treats sketches in their entirety (algorithm 1), supersampler focuses on small related partitions. One partition corresponding to every super-*k*-mers sharing a common minimizer. This allows for two distinct computational improvements. First, a partition that is specific to a file can be skipped as we know that no *k*-mer present in such partition will be found in another file so no matching *k*-mer exists for this partition, e.g. a minimizer not seen anywhere else. Second, the size of the partitions stored in memory being small, we expect few cache-misses when comparing partitions unlike sourmash for which a cache-miss for each queried fingerprint can be expected. See algorithm 2 for the sketch comparison for supersampler. In other words, as illustrated in Fig. 4, supersampler concentrates exclusively on small, relevant partitions and processes each of them only once. The efficiency benefits of this approach are magnified when comparing one or multiple documents against a large collection, as supersampler processes only a specific partition of the relevant documents at a given time. This targeted processing reduces the computational load and enhances the overall performance of the comparison.

**Algorithm 1:**
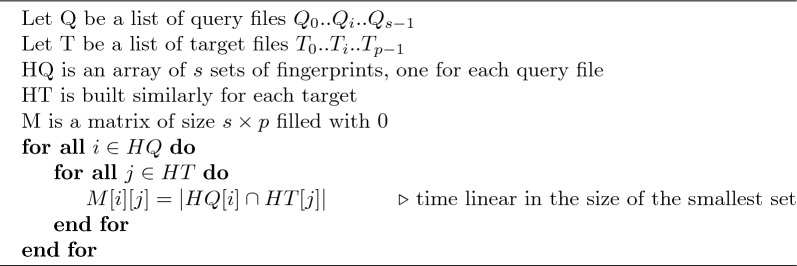
Sketch comparison in sourmash

**Algorithm 2:**
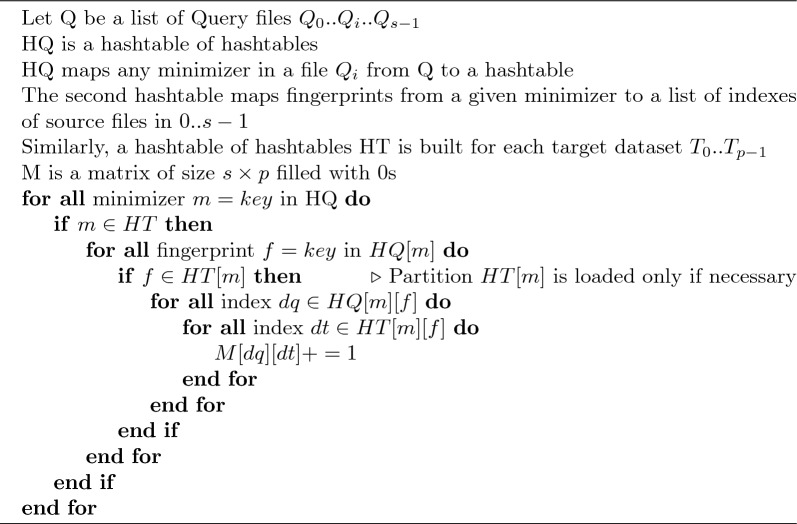
Sketch comparison in supersampler

### Multiset comparisons

*Angular similarity*

When considering the multiplicities of *k*-mers, the similarity between two *k*-mers multisets can be determined using cosine similarity, which is derived from the Euclidean dot product formula. This metric has already been used in sourmash and *simka*. Simka’s authors showed this distance was strongly correlated to taxonomic distance ( [25] page 15-17). it then felt logical to add it in supersampler to offer the same range of computations as sourmash.

The cosine similarity between vectors $A {\ = \lbrace A_1, \dots , A_n \rbrace }$ and $B {\ = \lbrace B_1, \dots , B_n \rbrace }$ is described by:

2 $$\begin{aligned} CS_{A,B}= \cos (\theta ) = \frac{A \cdot B}{|A||B|}= \frac{\sum _{i=1}^{n} A_i B_i}{\sqrt{\sum _{i=1}^{n} A_i^2\sum _{i=1}^{n} B_i^2}} \end{aligned}$$

Often, the term “cosine distance” is introduced:

3 $$\begin{aligned} CD_{A,B}=1-CS_{A,B} \end{aligned}$$

However, it is noteworthy that the cosine distance is not a true distance metric because it does not adhere to the triangle inequality. To acquire the triangle inequality property, one can use the angular distance:

4 $$\begin{aligned} AD_{A,B} = \frac{\arccos \left( CS_{A,B}\right) }{\pi } \end{aligned}$$

Thus, angular similarity becomes:

5 $$\begin{aligned} AS_{A,B}=1-AD_{A,B} \end{aligned}$$

In supersampler, we’ve introduced a specific mode to compute angular similarity. A distinguishing feature of this mode is that it counts the abundances of fingerprints using a memory-efficient hashmap based on robinhood hashing https://github.com/martinus/unordered_dense. Because each *k*-mer in the sketch is associated with its abundance, it results in a greater disk space usage as reported in Table 5. During the comparison stage, this information allows for the computation and display of Jaccard, Containment, and Angular similarities

*Reducing space overhead* Storing an integer for every *k*-mer can be resource-intensive, especially for large sketch collections. To mitigate this, we introduce two heuristic methods designed to drastically reduce overhead, yet still offer a reasonable approximation of angular distance. Given that applications such as metagenomics and RNA-seq can exhibit abundance variations spanning several orders of magnitude, precise abundance values are not always crucial. Instead, logarithmic abundances are more commonly examined. Consequently, we offer a mode that stores log abundances, which substantially decreases disk usage by compressing minuscule values. Log abundances are computed by simply applying a log scaling of the abundance counts for each *k*-mer. Another strategy is to retain just one abundance value for each super-*k*-mer, representing the average abundance of its constituent *k*-mers. This technique slashes the number of integers stored by a significant factor as showed in Table 5.

**Fig. 5:**
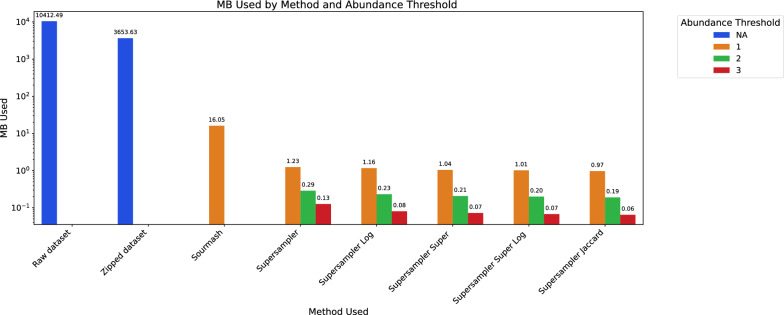
Comparison of different sketch heuristics and base storage of a 100X simulated C. elegans reads. Sourmash and Supersampler represent regular *k*-mer abundance encodings, while ‘Log’ represents a logarithmic *k*-mer abundance encoding. ‘Super’ is used to denote super *k*-mer abundance encoding, ‘Super log’ denotes super *k*-mer logarithmic abundance encoding, and ‘Jaccard’ indicates the absence of abundance encoding

Comparisons have been made using a 100X simulated sequencing with a 1% substitution rate of *C. elegans* with a subsampling rate of 1000. The table compares the size of storing the whole dataset raw and zipped versus the dataset subsampled with several heuristics detailed earlier. It shows that sourmash’s sketch stored with abundance uses 16Mbytes in memory when supersampler’s equivalent uses around 1Mbyte. It then shows the evolution of memory consumption with different manners of storing *k*-mers and super-*k*-mers abundances with several *k*-mer abundance filters.

## Results

All scalability and performance experiments were performed on a single cluster node running with Intel(R) Xeon(R) Gold 6130 CPU @ 2.10GHz and 2 64GiB DIMM DDR4 Synchronous 2666 MHz ram. Experiments about disk consumption and error for Angular similarity were performed on a HP elitebook running with Intel(R) core i7 12th generation with 32Gb RAM.

In the first section, we evaluate supersampler sketches space usage. In the second section, we evaluate the precision and performance of supersampler in comparison to sourmash, the current state-of-the-art solution. Finally, in the last section, we demonstrate supersampler’s scalability when indexing extensive collections.

### Space efficiency of supersampler

As previously discussed, the lower bound of memory cost for storing *k*-mers as super-*k*-mers is given by

$$2 \cdot \frac{3k - m - 1 + \log _2(k - m + 1)}{k - m + 1}$$

bits per *k*-mer, assuming we store the size of each super-*k*-mer in the index. For $m = 15$, this bound equates to 9.2 bits/*k*-mer with $k=31$ and 7.1 bits/*k*-mer with $k = 63$. However, in practice, supersampler exhibits lower space usage. Figure 16 in the supplementary materials reveals that approximately 6.5 bits/*k*-mer and 5 bits/*k*-mer are achieved for $k = 31$ and 63, respectively.

**Fig. 6:**
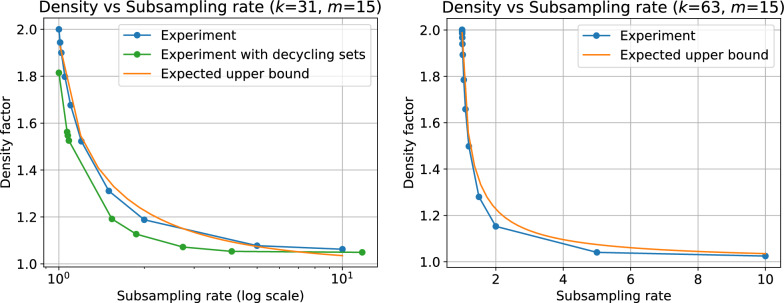
Measured density factor compared to the model

These results can be attributed to the low density permitted by supersampler’s minimizer selection scheme. As illustrated in Fig. 6, the density factor quickly diminishes as the subsampling rate increases, respectively as the fraction f diminishes. When the subsampling rate is 2, the density factor falls below 1.5, the lower bound of the minimizer scheme, and continues to decline toward 1. In general, subsampling tools seldom apply rates below 100, with sourmash defaulting to a rate of 1000 i.e. $f = 1/1000$. Consequently, supersampler consistently remains close to the lower bound for the density factor, since the density factor for a subsampling rate of 100 is already below 1.04. This facilitates the indexing of longer super-*k*-mers, which are stored more efficiently as their length increases.

**Fig. 7:**
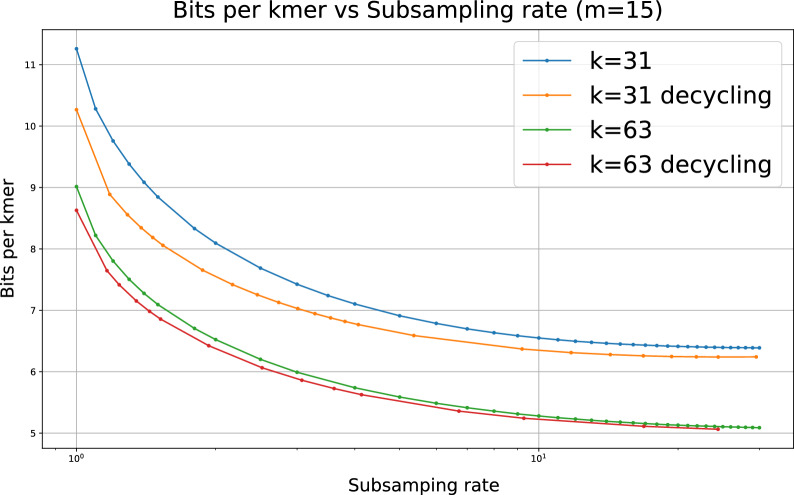
Space cost in bits per *k*-mer according to the subsampling rate with and without using decycling sets (yellow and red lines)

To further reduce memory costs, supersampler offers an option to use its selection scheme in conjunction with existing UHS-based minimizer schemes, specifically the modified double decycling sets introduced in [23]. As depicted in Fig. 7, this approach marginally improves the bit/*k*-mer cost, particularly for smaller values of *k*.

**Fig. 8:**
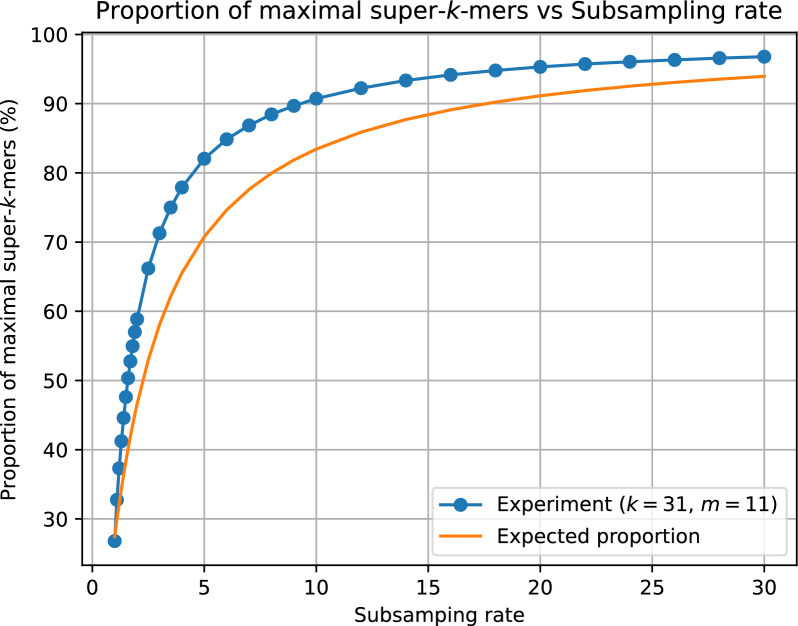
Fraction of maximal super-*k*-mers according to the subsampling rate

A high proportion of maximal super-*k*-mers in a sketch is advantageous for supersampler, as it lowers the bit/*k*-mer cost. Figure 8 demonstrates that the percentage of maximal super-*k*-mers increases rapidly with the subsampling rate, reaching 90%, 99%, and 99.9% of indexed super-*k*-mers when the subsampling rate is around 10, 100, and 1000, respectively. This feature is particularly significant because it enables a rapid and considerable reduction in the bit/*k*-mer cost by efficiently encoding maximal super-*k*-mers. Therefore, with the subsampling rates commonly used in practice, which involve a very high proportion of maximal super-*k*-mers, the actual bound is determined by the following formula

$$\frac{2(2k-m)}{k-m+1}$$

### Performance comparison

In our qualitative experiment, we compared the performance of supersampler with sourmash, which implements FracMinHash. We evaluated both tools on two distinct datasets: 1024 Salmonella genomes from GenBank and 1024 bacterial genomes from RefSeq. These collections were chosen due to their differing containment indexes; Salmonella genomes are highly similar to each other, while the bacterial genomes in RefSeq exhibit much greater dissimilarity (Jaccard similarity close to 0).

We carried out an all-versus-all comparison of these collections using both tools and monitored RAM and disk usage, as well as computation time during the sketch comparisons. To assess the precision of the approximated scores, we calculated the exact Jaccard and containment similarity values using Simka [25], which performs efficient *k*-mer counting operations on large collections. With these scores as a reference, the precision of the approximation can be evaluated.

RAM and computation time were measured using the benchmark flag from Snakemake, with one run per command. Disk usage was determined by comparing the sketch sizes of sourmash (using the *zip* option for *sourmash sketch*) and supersampler, with the latter’s sketch sizes examined through a Python script. supersampler sketches were stored in a tar archive and compressed using gzip -9.

**Fig. 9:**
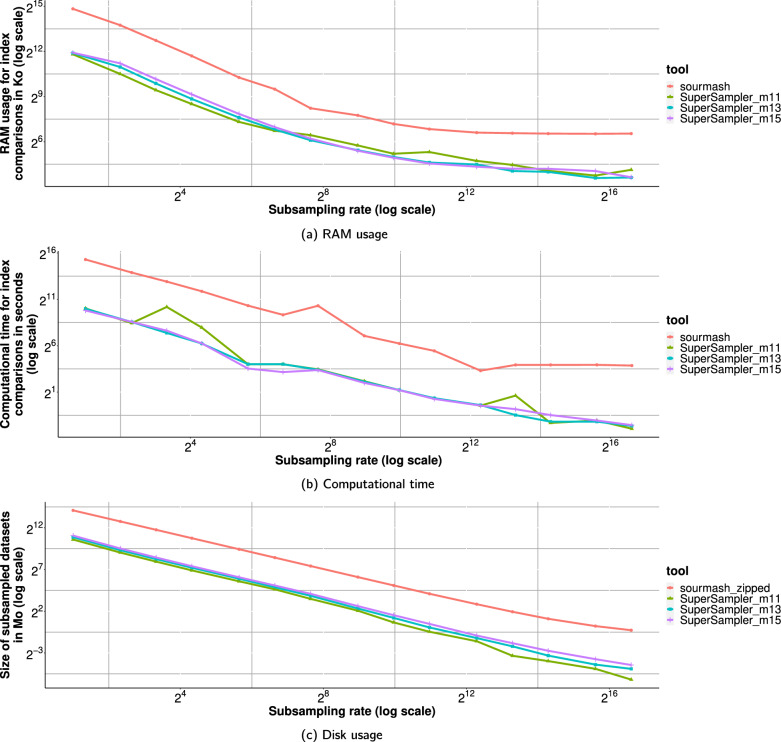
Resource consumption results for 1024 bacterial genomes from RefSeq. For these results, $k = 31$. For results on Salmonella genomes and $k = 63$, see Figures 17, 18,19 and in the appendix

#### RAM, time, and sketch size

Figures 9,17 and demonstrate that, for $k=31$, supersampler generally consumes 5 times less RAM and requires generally 16 times less space than sourmash. Additionally, supersampler performs computations 50 times faster than sourmash when comparing highly dissimilar genomes. However, when genomes are very similar, such as with Salmonella, comparison times are comparable since supersampler’s time optimization does not apply on very similar documents. We also note that minimizer size has little to no impact on these metrics.

Figures 18 and 19 reveal that the improvement in sketch disk size is even more significant with larger values of *k*. supersampler uses in general 50 times less disk space than sourmash with $k=63$, while maintaining similar differences in RAM and computation time.

#### Error

**Fig. 10:**
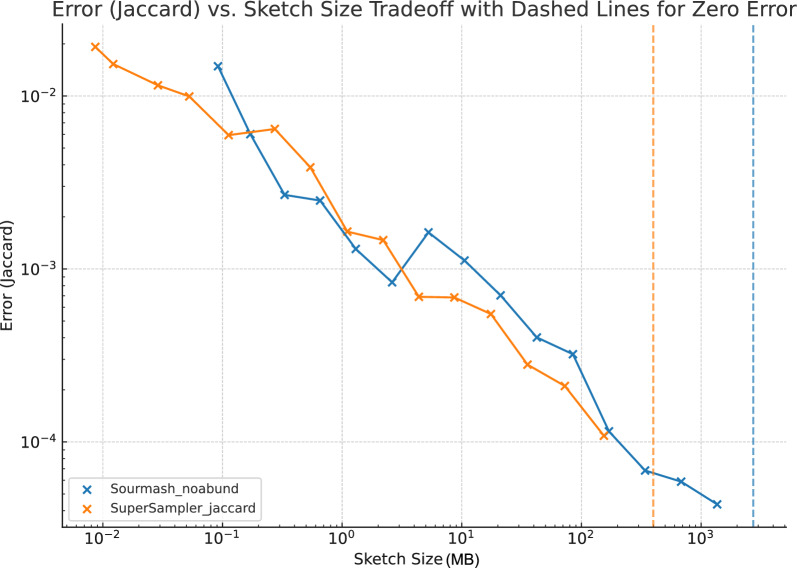
Error Jaccard similarity approximation for sourmash and supersampler against the compressed sketch sizes. This plot shows the results for 20 files of salmonellas simulated reads with $k = 31$, $m = 13$. Reads are 150bp long for a 100X coverage. Dashed lines represent the sizes of the sketches indexing all *k*-mers

**Fig. 11:**
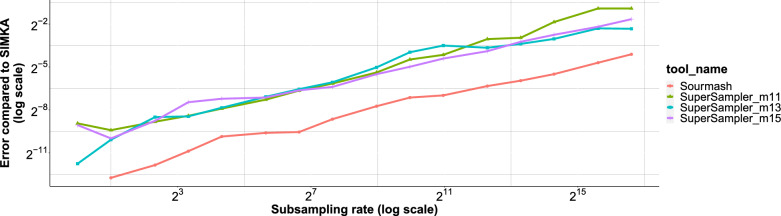
Error against Simka on Containment similarity approximation for sourmash (red line) and supersampler with different minimizer sizes. This plot is for 1024 Salmonellas genomes with $k = 63$. Other results for RefSeq and Salmonellas are available at Figure 20. Jaccard similarity error is available at Figure 21 in the appendix

As depicted in Fig. 10, supersampler’s error performance is on par with sourmash, though it does display a marginally lower accuracy. This slight dip in accuracy can be attributed to a clustering effect, which arises when supersampler selects overlapping *k*-mers around small minimizers. However, this effect is offset by supersampler’s proficiency in indexing and comparing a larger number of *k*-mers using equivalent memory and typically in a shorter computation time. In Fig. 11, we further assess the trade-off between accuracy and compressed sketch size by measuring the error relative to sketches compressed at gzip’s maximum compression level. Our findings indicate that, in terms of the precision-to-cost ratio, supersampler holds a favorable position against sourmash.

Additionally, supersampler stores *k*-mers in plain text without any loss of information, which means that *k*-mers of interest can actually be retrieved. While sourmash could rely on invertible hash functions and larger hashes to match this ability, doing so would effectively increase their space usage.

#### Massive collection indexing

**Fig. 12:**
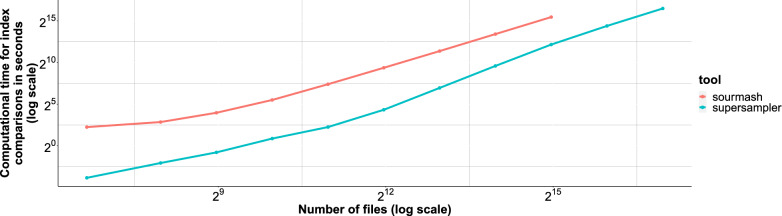
Computational time for comparisons on different amounts of bacterial genomes from RefSeq. From 100 to 128,000 genomes with $k = 63$, $s = 1000$ and $m = 15$ sourmash was run up to 32,000 genomes as it was taking too much time for the 2 last experiments

As a scalability experiment, supersampler and sourmash were monitored on their performances while analysing growing collection of RefSeq bacterial genomes. Result are displayed in Fig. 12. We can see that our tool is able to handle all versus all comparison very large scale collection comparison effectively. For the biggest amount of files, supersampler took 25 CPU hours. We observe that the gap between sourmash and supersampler is diminishing for larger collection as the output matrix itself become large and generate cache-miss for every update. The sketch creation step is essentially cheap as both tools only took a couple CPU hours the actual bottleneck being IO.

#### Angular similarity

To benchmark our tool directly against the angular similarity estimator of sourmash, we simulated 20 read sets, each 150bp in length, from 20 Salmonella genomes at a 100X coverage with a 0.1% error rate. We then evaluated each tool based on the deviation in their estimations from the original, unsubsampled sets. This provided insight into how each tool’s accuracy varied as the subsampling rate increased. It is worth noting that while SPSP can apply an abundance cutoff, we did not utilize this feature in our experiment to ensure a fair comparison with sourmash, which lacks this capability. Thus, any *k*-mer could be selected in our tests, irrespective of its abundance.

**Fig. 13:**
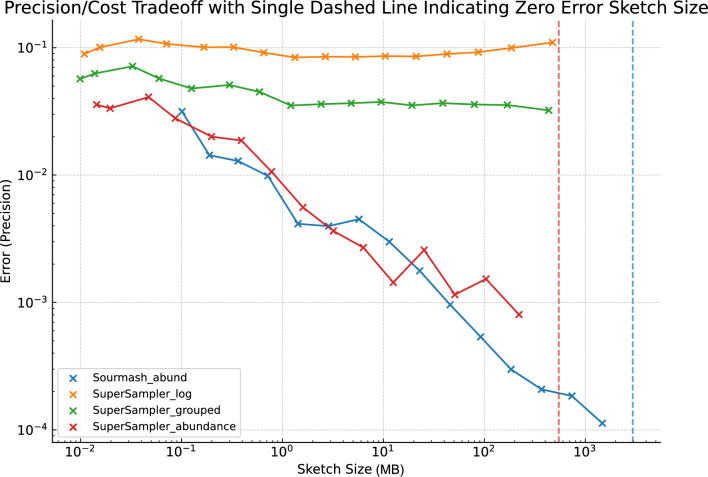
Error on Angular similarity approximation for sourmash and the different supersampler strategies. This plot shows the results for 20 files of salmonellas simulated reads with $k = 31$, $m = 13$. Reads are 150bp long for a 100X coverage. Dashed lines represent the sizes of the sketches indexing all *k*-mers

We observe in Fig. 13 that whilst SPSP is still less precise than sourmash, the gain in disk, time and RAM is largely compensating for this loss of precision, even when taking abundances into account.

## Conclusion

In this paper, we present both theoretical and practical results of an innovative subsampling scheme based on super-*k*-mers. We introduce the fractional hitting sets framework and propose a straightforward sketching method to highlight its benefits. This approach offers improved density compared to other schemes and tends to select *k*-mers that contribute to better space usage. Capitalizing on this scheme, we propose supersampler, an open-source sketching method for metagenomic assessment.

Through comprehensive experimental evaluation, we demonstrate that supersampler enables efficient and lightweight analysis of extensive genomic data sets with fewer resource requirements compared to the state-of-the-art tool sourmash. More generally our results confirm the validity of our methodology from both theoretical and experimental standpoints.

We recognize several potential enhancements for our study. First, concerning supersampler’s implementation, we aim to refine the tool for increased user-friendliness and adaptability for routine analysis while augmenting its capabilities. Implementation of such improvements will lead to more thorough experiments with existing sampling methods [26] as well as new comparisons with sourmash using the same amount of disk memory in order to better show supersampler’s capacity with regard to both fixed-size and scalable sketches. Other, more distant methods and different metrics such as ANI are used to answer the same problems. Comparing supersampler to such metrics and methods is the next step to further support supersampler’s interest.

We plan to investigate alternative methods for sketch comparison, like sorted fingerprints, which could potentially reduce the complexity of the comparison process. From a theoretical perspective, delving deeper into the properties of Fractional Hitting Sets and gaining a better understanding of density and restricted density bounds for various values of *f* may lead to even more efficient and robust sketching techniques.

## Figures and Tables

**Fig. 14:**
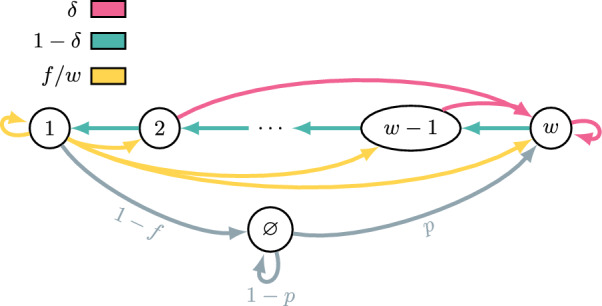
The chain is in state $i \in \llbracket 1, w \rrbracket$ if the small minimizer starts at position $i$ in the $k$-mer, and $\varnothing$ if there is no small minimizer. Different edge colors represent different probabilities

**Fig. 15:**
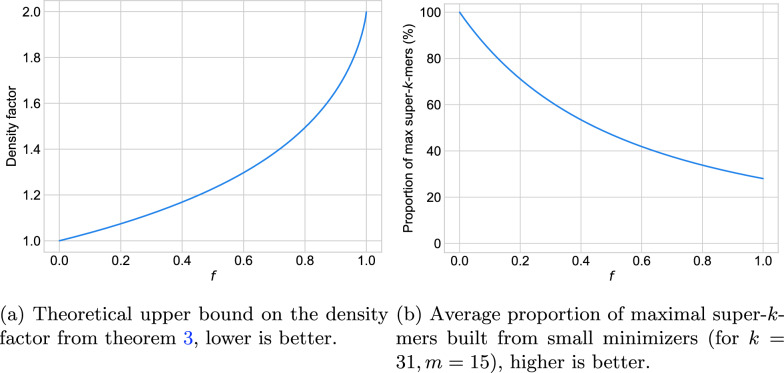
Theoretical bounds on the density factor and the proportion of maximal super-*k*-mers depending on the fraction *f* of covered *k*-mers

**Fig. 16:**
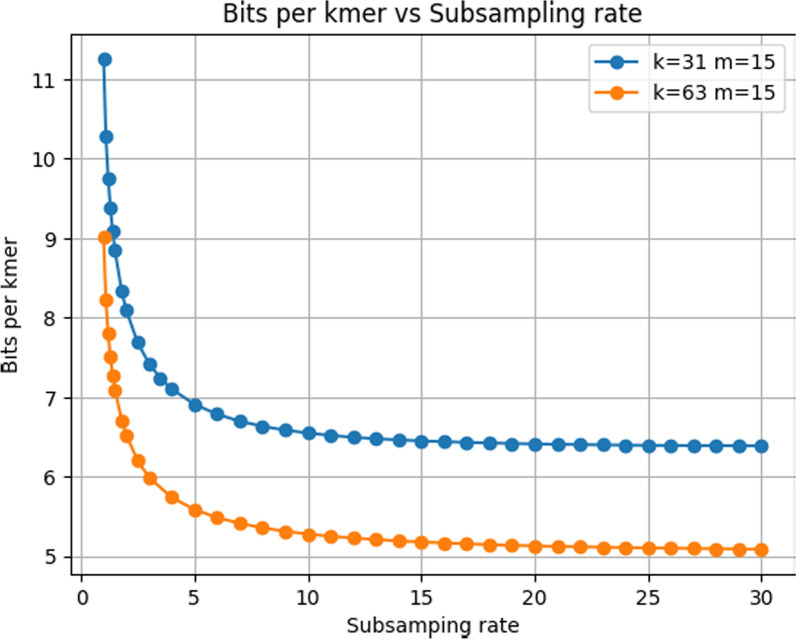
Space cost in bits per *k*-mer according to the subsampling rate

**Fig. 17:**
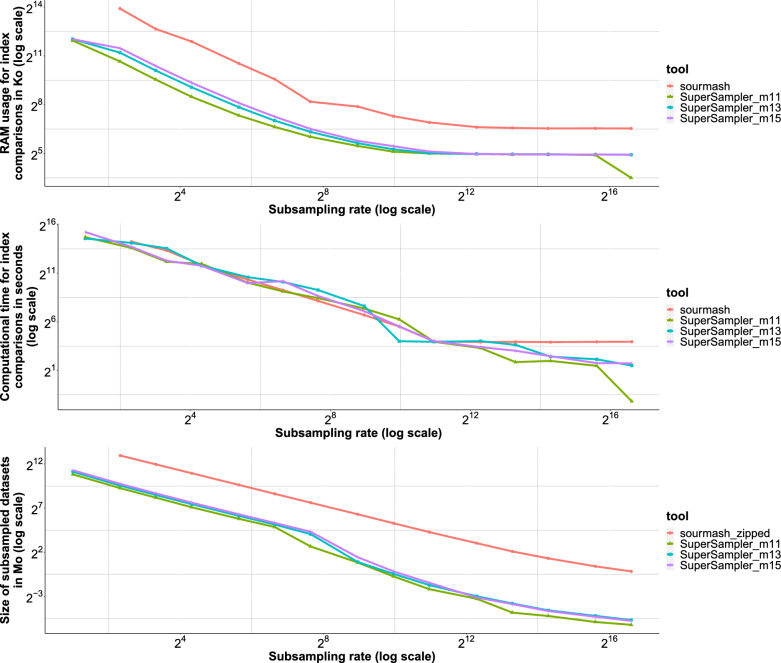
Salmonellas 1K k=31

**Fig. 18:**
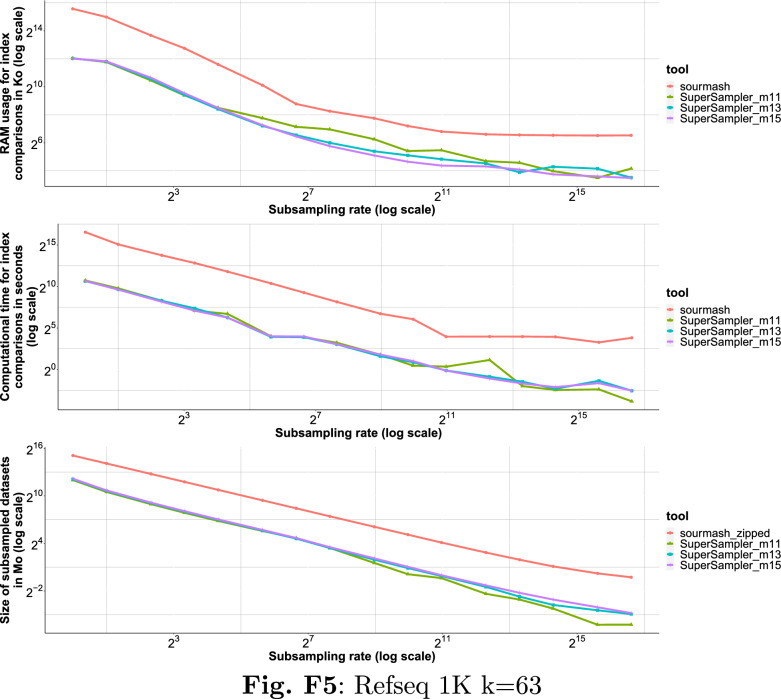
Refseq 1K k=63

**Fig. 19:**
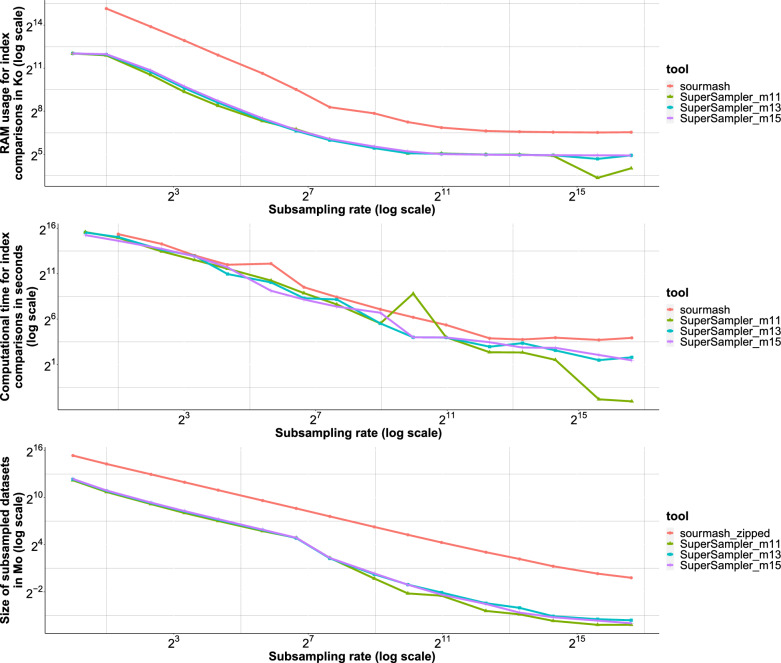
Salmonellas 1K k=63

**Fig. 20:**
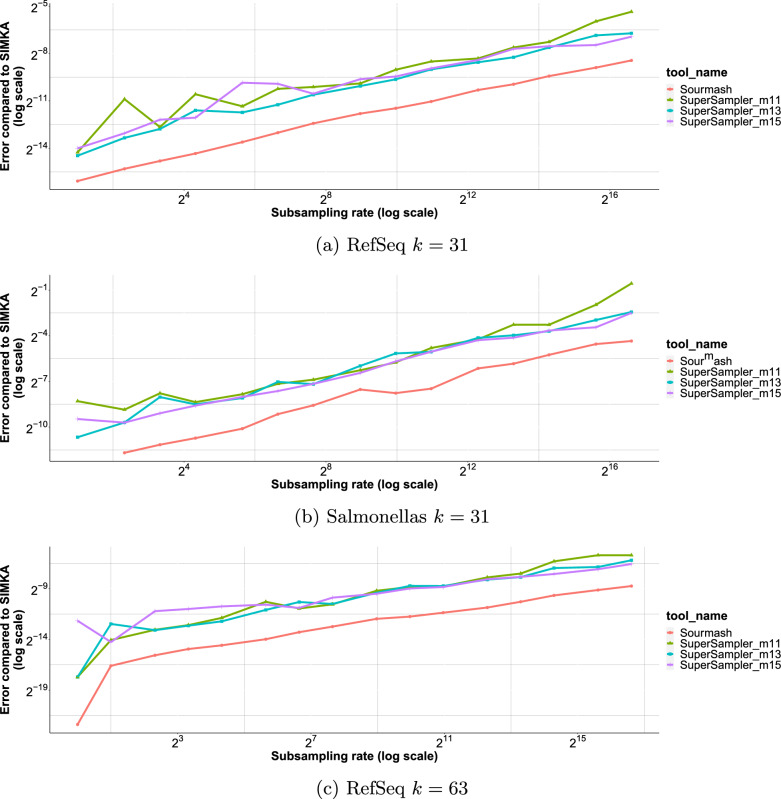
Error for containment similarity approximation for sourmash (red line) and supersampler on different values for minimizer sizes

**Fig. 21:**
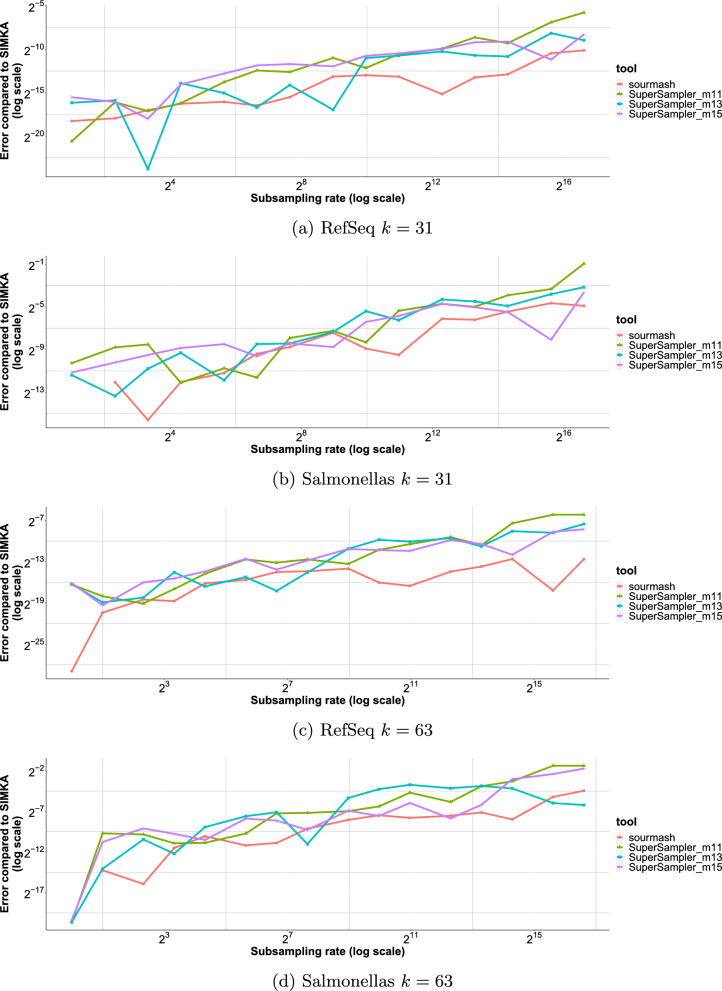
Error for Jaccard similarity approximation for sourmash (red line) and supersampler on different values for minimizer sizes

## Data Availability

Every reference genomes accession number, scripts and the precisions about the methodology for the experiments led for this research can be found on GitHub: https://github.com/TimRouze/supersampler/experiments. The code for supersampler is available on GitHub: https://github.com/TimRouze/supersampler.

## Appendix 1

### Useful lemmas

#### Lemma 1

Assuming $p$ is non-increasing with respect to $w$, $1 - (1 - p)^{w+1} \underset{w \rightarrow \infty }{=}\ f + o(1)$

#### Proof

$$(1-p)^{w+1} = (1-p)^w - p (1-p)^w$$

- if $$p \mathop {\longrightarrow }\limits _{w \rightarrow \infty } 0$$, $$p (1-p)^w \mathop {\longrightarrow }\limits _{w \rightarrow \infty } 0$$
- otherwise $p \ge c$ for some $c> 0$ since it is non-increasing, so $p (1 - p)^w \le p (1 - c)^w \mathop {\longrightarrow }\limits _{w \rightarrow \infty } 0$

Therefore, $$(1-p)^{w+1} = (1-p)^w + o(1)$$ $$\square$$

We use $\widehat{\mathcal {S}}$ to denote the set of $k$-mers containing a small $m$-mer.

#### Lemma 2

Given two consecutive *k*-mers $W_0$ and $W_1$, $\mathbb {P}(W_0, W_1 \in \widehat{\mathcal {S}}) \underset{w \rightarrow \infty }{=}\ f + o(1)$

#### Proof

$$\begin{aligned} \mathbb {P}(W_0, W_1 \in \widehat{\mathcal {S}})&= 1 - \mathbb {P}(W_0 \notin \widehat{\mathcal {S}}\vee W_1 \notin \widehat{\mathcal {S}}) \\&= 1 - \left[ \mathbb {P}(W_0 \notin \widehat{\mathcal {S}}) + \mathbb {P}(W_1 \notin \widehat{\mathcal {S}}) - \mathbb {P}(W_0 \notin \widehat{\mathcal {S}}\wedge W_1 \notin \widehat{\mathcal {S}}) \right] \\&= 1 - 2 (1 - p)^w + (1 - p)^{w+1} = 1 - (1 - p)^w + o(1) \qquad ({\text {Lemma}}~1) \\&= f + o(1) \end{aligned}$$

$$\square$$

#### Lemma 3

$$p \underset{w \rightarrow \infty }{=}\ -\frac{1}{w} \ln (1 - f) + o(1/w)$$

#### Proof

Because of property 1, we have $p = 1 - (1 - f)^{1/w}$ and

$$(1 - f)^{1/w} = \exp \left[ \frac{1}{w} \ln (1 - f) \right] = 1 + \frac{1}{w} \ln (1 - f) + o(1/w)$$

$$\square$$

## Appendix 2

### Proof of theorem 1

In order to upper bound the density, we follow the same approach as the one presented in [22] (for the proof of theorem 7). As stated in [22], the density is equivalent to the probability that a context *c* (that is, the string formed by two consecutive *k*-mers) is *charged*, i.e. the two *k*-mers of *c* have different minimizers.

$$\begin{aligned} d&= \mathbb {P}_{c, h}(c \text { is charged}) \\&\le \mathbb {P}_{c, h}(c \text { has duplicate } m-\text {mers}) + \mathbb {P}_{c, h}\left( c \text { is charged}\ |\ \text {no duplicate} m \text {-mers} \right) \end{aligned}$$

#### Lemma 4

(lemma 9 from [22]) Assuming $m> (3 + \varepsilon ) \log _\sigma w$,

$$\begin{aligned} \mathbb {P}_{c, h}(c \text { has duplicate} m-\text { mers}) = o(1 / w) \end{aligned}$$

If $c$ has no duplicate $m$-mers, the small $m$-mers are all distinct and each of them has the same probability to be minimal since $h$ is random. Therefore,

$$\begin{aligned} \mathbb {P}_{c, h}\left( c \text { is charged}\ |\ \text {no duplicate } m \text {-mers} \right) = \mathbb {E}_{c, h}\left[ \frac{M_{boundary}}{M_{total}} \right] \end{aligned}$$

where $M_{boundary}$ denotes the number of boundary $m$-mers that are small and $M_{total}$ denotes the total number of small $m$-mers in $c$.

Let $x_0$ denote the first $m$-mer of $c$ and $x_w$ denote the last one,

$$\begin{aligned} \mathbb {E}_{c, h}\left[ \frac{M_{boundary}}{M_{total}} \right]&= \mathbb {E}_{c, h}\left[ \frac{\textbf{1}_{x_0 \in \mathcal {S}} + \textbf{1}_{x_w \in \mathcal {S}}}{M_{total}} \right] = 2 \cdot \mathbb {E}_{c, h}\left[ \frac{\textbf{1}_{x_0 \in \mathcal {S}}}{M_{total}} \right] \qquad {\text {symmetry}} \\&= 2 \cdot \mathbb {E}_{c, h}\left[ 1 / M_{total}\ |\ x_0 \in \mathcal {S} \right] \cdot \mathbb {P}(x_0 \in \mathcal {S}) \end{aligned}$$

Assuming $x_0$ is small, we have $M_{total} = 1 + X$ with $X \sim B(w, p)$, since each other $m$-mer of $c$ has a probability $p$ to be small.

$$\begin{aligned} \mathbb {E}_{c, h}\left[ 1 / M_{total}\ |\ x_0 \in \mathcal {S} \right]&= \mathbb {E}_{c, h}\left[ \frac{1}{1 + X} \right] = \sum _{i = 0}^w \frac{1}{1 + i} \left( {\begin{array}{c}w\\ i\end{array}}\right) p^i (1-p)^{w-i} \end{aligned}$$

#### Lemma 5

$$\sum _{i = 0}^w \frac{1}{1 + i} \left( {\begin{array}{c}w\\ i\end{array}}\right) p^i (1-p)^{w-i} = \frac{1 - (1 - p)^{w+1}}{(w + 1) p}$$

Finally, since $$\mathbb {P}(x_0 \in \mathcal {S}) = p$$, $$d \le 2 \cdot \frac{1 - (1 - p)^{w+1}}{w + 1} + o(1/w) = \frac{2 f}{w + 1} + o(1/w) \quad \text {(Lemma}~1)$$

## Appendix 3

### Proof of theorem 2

In this section, **we assume that every** *k*-**mer we work with contains a small** $m$-**mer**.

Just as for the proof of theorem 1, we still have

$$d \le \mathbb {P}_{c, h}(c \text { has duplicate} m- \text {mers}) + \mathbb {P}_{c, h}\left( c \text { is charged}\ |\ \text {no duplicate} m \text {-mers} \right)$$ and

$$\begin{aligned} \mathbb {P}_{c, h}\left( c \text { is charged}\ |\ \text {no duplicate} m \text {-mers} \right)&= \mathbb {E}_{c, h}\left[ \frac{M_{boundary}}{M_{total}} \right] \\ &= 2 \cdot \mathbb {E}_{c, h}\left[ 1 / M_{total}\ |\ x_0 \in \mathcal {S} \right] \cdot \mathbb {P}(x_0 \in \mathcal {S}\ |\ W_1 \in \widehat{\mathcal {S}}) \end{aligned}$$

#### Lemma 6

Assuming $$m> (3 + \varepsilon ) \log _\sigma w$$, $$\mathbb {P}_{c, h}(c \text { has duplicate } m \text {-mers}) = o(1 / w)$$

#### Proof

This proof is similar to the proof of lemma 9 from [22]. Let $i, j \in \llbracket 0, w \rrbracket$ with $i < j$, $\delta = j - i$.

If $$\delta < m$$, $$\mathbb {P}(x_i = x_j) = \frac{\sigma ^\delta }{\sigma ^{m + \delta }} = \frac{1}{\sigma ^m} = o(1/w^3)$$

If $$\delta \ge m$$,

$$\begin{aligned} \mathbb {P}(x_i = x_j)&= \mathbb {P}(x_i = x_j\ |\ x_i, x_j \in \mathcal {S}) \mathbb {P}(x_i, x_j \in \mathcal {S}) + \mathbb {P}(x_i = x_j\ |\ x_i, x_j \notin \mathcal {S}) \mathbb {P}(x_i, x_j \notin \mathcal {S}) \\&= \frac{\mathbb {P}(x_i, x_j \in \mathcal {S})}{p \cdot \sigma ^m} + \frac{\mathbb {P}(x_i, x_j \notin \mathcal {S})}{(1-p) \sigma ^m} \end{aligned}$$

Because of lemma 2, $\mathbb {P}(x_i, x_j \in \mathcal {S}) = \frac{p^2}{\mathbb {P}(W_0, W_1 \in \widehat{\mathcal {S}})} = \frac{p^2}{f + o(1)} \le p$ and

$$\begin{aligned} \mathbb {P}(x_i, x_j \notin \mathcal {S}) \le \frac{(1-p)^2 \left[ 1 - (1-p)^{w-1} \right] }{\mathbb {P}(W_0, W_1 \in \widehat{\mathcal {S}})} = \frac{(1-p)^2 \left[ 1 - (1-p)^{w-1} \right] }{f + o(1)} \le (1-p)^2 \end{aligned}$$

Therefore, $$\mathbb {P}(x_i = x_j) \le \frac{p}{p \cdot \sigma ^m} + \frac{(1-p)^2}{(1-p) \sigma ^m} \le \frac{2}{\sigma ^m} = o(1/w^3)$$

Thus $$\mathbb {P}_{c, h}(c \text { has duplicate } m \text {-mers}) = \left( {\begin{array}{c}w\\ 2\end{array}}\right) \times o(1/w^3) = o(1/w)$$ $$\square$$

Assuming $x_0$ is small, the $w$ next $m$-mers of $c$ form a *k*-mer, so we know that at least one of them is also small. Therefore,

$$\begin{aligned}&\mathbb {E}_{c, h}\left[ 1 / M_{total}\ |\ x_0 \in \mathcal {S} \right] = \mathbb {E}_{c, h}\left[ \frac{1}{1 + X}\ |\ X \ge 1 \right] = \frac{1}{\mathbb {P}(X \ge 1)} \sum _{i = 1}^w \frac{1}{1 + i} \left( {\begin{array}{c}w\\ i\end{array}}\right) p^i (1-p)^{w-i} \\&= \frac{1}{f} \left[ \frac{1 - (1 - p)^{w+1}}{(w + 1) p} - (1 - p)^w \right] = \frac{1}{f} \left[ \frac{f + o(1)}{(w + 1) p} - (1 - f) \right] \qquad (\text {Lemma}~5 \text {and}~1) \end{aligned}$$

What’s more, $\mathbb {P}(x_0 \in \mathcal {S}\ |\ W_1 \in \widehat{\mathcal {S}}) = \frac{\mathbb {P}(x_0 \in \mathcal {S})}{\mathbb {P}(W_1 \in \widehat{\mathcal {S}})} = \frac{p}{f}$. Hence,

$$\begin{aligned} d&\le \frac{2 p}{f^2} \left[ \frac{f + o(1)}{(w + 1) p} - (1 - f) \right] + o(1/w) = \frac{2}{f (w+1)} - \frac{2 (1 - f) p}{f^2} + o(1/w) \\&= \frac{2}{f (w+1)} + \frac{2 (1 - f) \ln (1 - f)}{f^2 w} + o(1/w) \qquad \,(\text {Lemma}~3) \\&= 2 \cdot \frac{f + (1 - f) \ln (1 - f)}{f^2 (w+1)} + o(1/w) \end{aligned}$$

## Appendix 4

### Proof of theorem 3

In order to compute the proportion of maximal super-*k*-mers, we adapt the proof of theorem 4 from [20] (Fig. 14).

First, we introduce a similar Markov chain representing the position *X* of the small minimizer in the *k*-mer, with an extra state $\varnothing$ when there is no small minimizer.

We reuse the following notations introduced in [20]:

- $P_{lr}$ is the proportion of left-right-max (i.e. maximal) super-*k*-mers
- $P_l$ is the proportion of left-max super-*k*-mers
- $P_r$ is the proportion of right-max super-*k*-mers
- $P_n$ is the proportion of non-max super-*k*-mers

$$\forall i \in \llbracket 1, w-1 \rrbracket , \mathbb {P}(\text {first } X = i) = \mathbb {P}(X = 1) \cdot \frac{f}{w}$$

$$\begin{aligned} P_{lr} + P_r = \mathbb {P}(X = w) = \mathbb {P}(\text {first } X = w) = 1 - \sum _{i=1}^{w-1} \mathbb {P}(\text {first } X = i) = 1 - \mathbb {P}(X = 1) \cdot f \cdot (1 - 1/w) \end{aligned}$$

$$\begin{aligned} P_{lr} + P_l = \mathbb {P}(\text {last } X = 1) = \mathbb {P}(X = 1) + \mathbb {P}(\text {first } X = 1) = \mathbb {P}(X = 1) \cdot (1 + f/w) \end{aligned}$$

By symmetry, $P_l = P_r$, so $1 - \mathbb {P}(X = 1) \cdot f \cdot (1 - 1/w) = \mathbb {P}(X = 1) \cdot (1 + f/w)$

Therefore, $$\mathbb {P}(X = 1) = \frac{1}{1 + f}$$ and $$\mathbb {P}(X = w) = 1 - \frac{f}{1 + f} \left( 1 - \frac{1}{w} \right) = \frac{1 + f/w}{1 + f}$$

What’s more, $1 + P_{lr} = P_{lr} + P_l + P_{lr} + P_r + P_n = 2 \cdot \mathbb {P}(X = w) + P_n$ so

$$\begin{aligned} P_{lr} = P_n + 2 \cdot \mathbb {P}(X = w) - 1 = P_n + \frac{1 - f(1 - 2/w)}{1 + f} \text { and } P_l = P_r = \mathbb {P}(X = w) - P_{lr} = \frac{f(1 - 1/w)}{1 + f} - P_n \end{aligned}$$

$$\begin{aligned} P_n&= \mathbb {P}(\text {first } X \ne w) \cdot \mathbb {P}(\text {last } X \ne 1) = \mathbb {P}(X = 1) \cdot f \cdot (1 - 1/w) \cdot \left[ 1 - \mathbb {P}(X = 1) \cdot (1 + f/w) \right] \\&= \left( 1 - \frac{1}{w} \right) \cdot \frac{f}{1 + f} \cdot \left[ 1 - \frac{1 + f/w}{1 + f} \right] = \left( 1 - \frac{1}{w} \right) \cdot \frac{f}{1 + f} \cdot \frac{f (1 - 1/w)}{1 + f} = \left[ \left( 1 - \frac{1}{w} \right) \frac{f}{1 + f} \right] ^2 \end{aligned}$$

Thus $$P_l = P_r = \left[ \left( 1 - \frac{1}{w} \right) \frac{f}{1 + f} \right] \left[ 1 - \left( 1 - \frac{1}{w} \right) \frac{f}{1 + f} \right]$$ and $$P_{lr} = \left[ \left( 1 - \frac{1}{w} \right) \frac{f}{1 + f} \right] ^2 + \frac{1 - f(1--2/w)}{1 + f}$$

## Appendix 5

### Proof of theorem 4

This proof generalizes the proof of theorem 1 when the minimizers are selected from a UHS $\mathcal {U}$ with density $d_\mathcal {U}$.

First, because of independence, we have $\mathbb {P}(x_0 \in \mathcal {S}\cap \mathcal {U}) = \mathbb {P}(x_0 \in \mathcal {S}) \cdot \mathbb {P}(x_0 \in \mathcal {U})$ and $\mathbb {P}(\left|\mathcal {S}\cap \mathcal {U}\cap c \right| = i) = \sum _{n \ge i} \mathbb {P}(\left|\mathcal {U}\cap c \right| = n) \mathbb {P}(\left|\mathcal {S}\cap \mathcal {U}\cap c \right| = i\ |\ \left|\mathcal {U}\cap c \right| = n)$

The main change of the proof lies in the bound on the expectation:

$$\begin{aligned} \mathbb {E}&\left[ \frac{1}{\left|\mathcal {S}\cap \mathcal {U}\cap c \right|}\ |\ x_0 \in \mathcal {S}\cap \mathcal {U} \right] = \sum _{i=0}^w \frac{1}{i+1} \mathbb {P}\left( \left|\mathcal {S}\cap \mathcal {U}\cap c \right| = i+1\ |\ x_0 \in \mathcal {S}\cap \mathcal {U} \right) \\&= \sum _{i=0}^w \frac{1}{i+1} \sum _{n=i}^w \mathbb {P}(\left|\mathcal {U}\cap c \right| = n+1\ |\ x_0 \in \mathcal {U}) \mathbb {P}(\left|\mathcal {S}\cap \mathcal {U}\cap c \right| = i+1\ |\ \left|\mathcal {U}\cap c \right| = n+1, x_0 \in \mathcal {S}\cap \mathcal {U}) \\&= \sum _{n=0}^w \mathbb {P}(\left|\mathcal {U}\cap c \right| = n+1\ |\ x_0 \in \mathcal {U}) \sum _{i=0}^n \frac{1}{i+1} \mathbb {P}(\left|\mathcal {S}\cap \mathcal {U}\cap c \right| = i+1\ |\ \left|\mathcal {U}\cap c \right| = n+1, x_0 \in \mathcal {S}\cap \mathcal {U}) \\&= \sum _{n=0}^w \mathbb {P}(\left|\mathcal {U}\cap c \right| = n+1\ |\ x_0 \in \mathcal {U}) \sum _{i=0}^n \frac{1}{i+1} \mathbb {P}(\left|\mathcal {S} \right| = i+1\ |\ x_0 \in \mathcal {S}, |W| = n) \\&= \sum _{n=0}^w \mathbb {P}(\left|\mathcal {U}\cap c \right| = n+1\ |\ x_0 \in \mathcal {U}) \cdot \frac{1 - (1-p)^{n+1}}{(n+1) p}\qquad (\text {Lemma}~5) \\&\le \sum _{n=0}^w \mathbb {P}(\left|\mathcal {U}\cap c \right| = n+1\ |\ x_0 \in \mathcal {U}) \cdot \frac{f + o(1)}{(n+1) p} \qquad (\text {Lemma}~1) \end{aligned}$$

Therefore, using the same arguments as in the proof of theorem 1, we obtain

$$\begin{aligned} d_{\mathcal {S}\cap \mathcal {U}}&\le 2 \cdot \mathbb {P}(x_0 \in \mathcal {S}\cap \mathcal {U}) \cdot \mathbb {E}\left[ \frac{1}{\left|\mathcal {S}\cap \mathcal {U}\cap c \right|}\ |\ x_0 \in \mathcal {S}\cap \mathcal {U} \right] + o(1/w) \\&\le 2 \cdot \mathbb {P}(x_0 \in \mathcal {U}) \cdot \mathbb {P}(x_0 \in \mathcal {S}) \sum _{n=0}^w \mathbb {P}(\left|\mathcal {U}\cap c \right| = n+1\ |\ x_0 \in \mathcal {U}) \cdot \frac{f}{(n+1) p} + o(1/w) \\&= f \cdot 2 \cdot \mathbb {P}(x_0 \in \mathcal {U}) \sum _{n=0}^w \frac{1}{n+1} \mathbb {P}(\left|\mathcal {U}\cap c \right| = n+1\ |\ x_0 \in \mathcal {U}) + o(1/w) \\&= f \cdot d_\mathcal {U}+ o(1/w) \end{aligned}$$
